# Genomic instability and DNA replication defects in progeroid syndromes

**DOI:** 10.1080/19491034.2018.1476793

**Published:** 2018-06-23

**Authors:** Romina Burla, Mattia La Torre, Chiara Merigliano, Fiammetta Vernì, Isabella Saggio

**Affiliations:** a Dipartimento di Biologia e Biotecnologie “C. Darwin”, Sapienza Università di Roma, Roma, Italy; b Istituto di Biologia e Patologia Molecolari del CNR, Rome, Italy; c Istituto Pasteur Fondazione Cenci Bolognetti, Rome, Italy

**Keywords:** Lamin, DNA replication, progeria, nuclear lamina, aging, DNA damage

## Abstract

Progeroid syndromes induced by mutations in lamin A or in its interactors – named progeroid laminopathies – are model systems for the dissection of the molecular pathways causing physiological and premature aging. A large amount of data, based mainly on the Hutchinson Gilford Progeria syndrome (HGPS), one of the best characterized progeroid laminopathy, has highlighted the role of lamins in multiple DNA activities, including replication, repair, chromatin organization and telomere function. On the other hand, the phenotypes generated by mutations affecting genes directly acting on DNA function, as mutations in the helicases WRN and BLM or in the polymerase polδ, share many of the traits of progeroid laminopathies. These evidences support the hypothesis of a concerted implication of DNA function and lamins in aging. We focus here on these aspects to contribute to the comprehension of the driving forces acting in progeroid syndromes and premature aging.

Progeroid syndromes are rare genetic diseases characterized by reduced lifespan and by premature appearance of symptoms related to physiological aging. For a majority of progeroid syndromes the causative mutation has been identified pointing to the role of DNA function and of lamins in these diseases. We report here on these syndromes, on their genetics and on the molecular mechanisms involved in generating the related phenotypes, focusing on the interrelationships between lamins and DNA function.

## Mutations affecting DNA function cause progeroid syndromes

Given the direct connection between DNA function and cell senescence and the implication of senescence in aging[1], it is not surprising that many progeroid syndromes are caused by mutations in genes encoding for DNA repair and DNA maintenance enzymes (Tables 1 and 2). These include the Werner Syndrome caused by mutations in the DNA helicase *WRN*[2], the Bloom Syndrome caused by mutation in the helicase *BLM* [3], the Rothmund-Thomson syndrome caused by mutations in the gene *RECQL4* [4], another DNA helicase, the Cockayne syndrome involving either the *ERCC8* or *ERCC6* genes, that are implicated in DNA repair [5], the Xeroderma pigmentosum also caused by mutations in the DNA repair machinery elements [6], the Dyskeratosis congenita involving genes implicated in telomere function, including *TERT, TERC* and *DKC1* [7], and the mosaic variegated aneuploidy syndrome caused by genes implicated in cell division[8].

**Table 1.** Progeroid syndromes caused by lamin and DNA function linked genes. The table highlights common characteristics of genetically different progeroid syndromes. The colored map indicates that progeroid traits are prevalently modulated in the same direction in progerias generated by either lamin (*LMNA, ZMPSTE24*) or DNA function (*POLD1, SPRTN, AKTIP*) mutations. hm: homozygous; hz: heterozygous; AD: autosomic dominant; AR: autosomic recessive; *de novo*: sporadic mutation; RJALS: Ruijs-Alfs syndrome; SCC: squamous cells carcinoma; HCC: hepatocellular carcinoma; H: hypomorphic mutation; kof: knock out first; * model organism mouse.

**Table 2.** Dissection of DNA functions impaired in progeroid syndromes of different origins. Alterations in one or more of the DNA functions, caused by mutations in the genes indicated, result in a premature aging phenotype. DC: Dyskeratosis congenita; WS: Werner syndrome; BS: Bloom syndrome; R-TS: Rothmund-Thomson syndrome; CS: Cockayne syndrome; XP: Xeroderma pigmentosum. Nd: not determined.

		DNA functions altered in progeroid syndromes					
Disease	Altered gene	Telomere metabolism	Chromatin remodeling	DNA transcription	DNA replication	DNA repair	References
HGPS, MADA, APS, AWS	*LMNA*	altered	altered	altered	altered	altered	[15,23,29,50,104]
DC	*TERT/TERC*	altered	nd	nd	nd	nd	[7]
DC	*DKC1*	altered	nd	nd	nd	nd	[7]
-	*AKTIP*	altered	altered	nd	altered	nd	[63,65]
WS	*WRN*	altered	altered	altered	altered	altered	[105–109]
BS	*BLM*	altered	altered	altered	altered	altered	[110–113]
R-TS	*RECQL4*	altered	nd	nd	altered	altered	[109]
CS	*ERCC8/ERCC6*	nd	nd	nd	nd	altered	[5]
RJALS	*SPRTN*	nd	nd	nd	altered	altered	[62,114]
MDPL	*POLD1*	nd	nd	nd	altered	altered	[70]
XP	*XPA*	nd	nd	nd	nd	altered	[31]

## Mutations of nuclear envelope components cause progeroid syndromes

Along with DNA function genes, a major genetic cause of progeroid syndromes are mutations of nuclear envelope factors or of their interactors [9], which defines the group of progeroid laminopathies. Differently from other premature aging syndromes, progeroid laminopathies have early onset, are usually pediatric diseases, and display more severe symptoms of aging. Another feature that distinguishes progeroid laminopathies from the other premature aging syndromes, is the absence of increased cancer susceptibility (Table 1).

The most characterized progeroid laminopathy is Hutchinson Gilford Progeria syndrome (HGPS), which is caused by a mutation in the *LMNA* gene generating a mutant lamin A indicated as progerin [10,11]. HGPS is an extremely rare, dominant genetic disorder affecting 1 in 8 million live newborns, with a prevalence of 1 in 20 million living individuals (The Progeria Research Foundation, 2018; https://www.progeriaresearch.org/prf-by-the-numbers/). Children affected by HGPS show severe growth retardation, alopecia, loss of subcutaneous fat, prominent eyes and scalp veins, thin skin, joint stiffness and reduced bone density. They suffer from osteoporosis, atherosclerosis and cardiovascular diseases, that finally cause their premature death at an average age of 14 years [12,13]. Patients with HGPS do not develop childhood tumors, and progerin exerts a tumor-protective function [9]. Along with this, cells from HGPS show different aberrant phenotypes including misshapen nuclei and a disorganized nuclear lamina and envelope [14], altered gene expression [15], cellular senescence [16], genomic instability, revealed by the persistent DNA damage response activation and defects in the repair pathways [17,18], impaired chromatin organization [19–21] and abnormal telomere metabolism [22,23]. This wide variety of aberrant phenotypic features likely reflects the high number of different, and essential, cellular processes in which lamin A is involved.

Restrictive Dermopathy (RD) is another example of lamin related progeroid syndrome. Its causative mutations are found in the gene *ZMPSTE24*, whose product is implicated in the maturation of lamin A. RD clinical phenotype differs from HGPS on that it is a perinatal lethal disease characterized by a severe intrauterine growth delay. After birth, affected children display a characteristic thin, translucent, tight skin, and a typical facial dysmorphism characterized by a small pinched nose, micrognathia and mouth in a fixed ‘o’ shape. RD patients suffer from joint contractures, respiratory insufficiency in which the respiratory failure most often leads to neonatal death within several weeks of birth [24].

Along with HGPS and RD, nuclear envelope related progerias include the Atypical Werner syndrome (AWS), the Atypical Progeria syndrome (APS), the MandibuloAcral Dysplasia type A (MADA), which have been all related to mutations in *LMNA*, and MandibuloAcral Dysplasia type B (MADB) which has been related to mutations in *ZMPSTE24*. MADA is a less severe progeroid laminopathy as compared to HGPS and RD. The clinical features appear at 4 years of age and become more severe with increasing age. This disorder is characterized by growth retardation, postnatal onset of craniofacial anomalies with mandibular hypoplasia, skeletal abnormalities with progressive distal phalanges and clavicular osteolysis. Some patients show progeroid features such as thin nose, sparse, brittle hair, and sclerodermatous skin. Among other typical features there are lipodystrophy and metabolic complications due to insulin resistance [25].

Another case of progeroid laminopathy, deserving particular attention in relation with DNA function, is the Nestor Guillermo Progeria syndrome (NGPS). NGPS is defined as chronic progeria because it has a slow clinical course and long survival despite its early onset. Patients display growth retardation, although less pronounced than HGPS patients, micrognatia, lipoatrophy and major skeletal deformation with severe osteoporosis and osteolysis [12,26]. NGPS is caused by mutations in *BANF1* gene encoding for the barrier-to-autointegration factor 1 (BAF1) protein. BAF1 binds the nuclear lamina related LEM proteins LAP2α, Emerin, and MAN1, and acts on DNA function by binding chromatin and histones [18,26,27]. NGPS is thus suggestive of a strong connection between nuclear lamina and DNA function in generating the progeroid phenotype.

isorder characterized by growth retardation, postnatal onset of craniofacial anomalies with mandibular hypo-plasia, skeletal abnorm alities with progressive distal phalanges, and clavicular osteolysis, in addition to skin changes like mottled pigmentati on and atrophy [Young et al., 1971; Freidenberg et al., 1992; Tudisco et al., 2000; Simha and Garg, 2002]. Some patients show progeroid (premature aging) features such as thin nose, sparse, brittle hair, and sclerodermatous (stiff and parched) skin. Among other typical features we can find lipodystrophy and metabolic complications due to insulin resistance and diabete

## Genomic instability is a common trait of progeroid syndromes

Genomic instability is a trait of progeroid syndromes deriving from both mutations in lamin and in factors controlling DNA function, and is emerging as driver of aging. In fact both physiological and premature aging can be read as the consequence of cell senescence, which, in turn is generated by genomic damage.

Progerin expressing samples accumulate DNA damage, which is highlighted by the presence of intranuclear γH2AX positive foci that are induced by DNA double strand breaks (DSBs). Foci increase in number with continued passage and, at the same time, cell growth rate slows down till reaching premature senescence [28,29]. Along with this, HGPS samples exhibit persistent DNA damage checkpoint activation, including the phosphorylation of the DNA damage signaling element ATM, the activation of the checkpoint regulators Chk1 and Chk2 and that of the guardian of the genome p53 [28,29]. Moreover, DNA repair is impaired in laminopathic cells. Fibroblasts from HGPS patients and mouse embryonic fibroblasts (MEFs) derived from progeroid models, such as *Zmpste24* knock out mice, are extremely sensitive to genotoxic agents as camptothecin and etoposide, which are DSBs inducers, but also to UV irradiation that typically activates the Nucleotide Excision Repair (NER) pathway [30]. This increased sensitivity is mechanistically explained by the observation that laminopathic cells display impaired recruitment of DNA repair proteins at DSBs, which then remain unrepaired. Indeed, an aberrant repair scheme has been observed in HGPS cells, characterized by delayed or inefficient recruitment of the DNA damage response factor 53BP1, of the Non Homologous End Joining (NHEJ) repair protein Ku70 and of the Homology directed repair (HDR) elements Rad50, Rad51, and by aberrant sequestration of the NER component XPA [31] at DSBs. These alterations block the correct DSB repair sequence and cause general impairment of NER [32].

## Altered protein-protein interaction bridges DNA function to nuclear envelope in progeroid syndromes

HGPS patients present *de novo* mutations in exon 11 of *LMNA* gene leading to the activation of a cryptic splice site that brings to the synthesis of progerin. Progerin does not contain the lamin A cleavage site for Zmpste24, for this reason the protein does not undergo the final step of processing, giving rise to a permanently farnesylated mutant protein. In a subset of progeroid laminopathies, in MADB and RD for example, the disease is caused by compound heterozygous and homozygous mutations in *FACE1* gene that encodes for Zmpste24. The mutations give rise to defective protein activity, total loss of function in RD, and lead to the accumulation of a prelamin A form that is not properly processed and retains the farnesylated C-terminus.

These results have suggested the idea that different progerias share a common functional cause in the accumulation of mutant forms of permanently farnesylated lamin A, that could be toxic for the cell. Based on this assumption, therapeutic approaches for progerias have been focused on drugs inhibiting the farnesylation of progerin and prelamin A, including lonafarnib, pravastatin and zoledronic acid [33]. However, the recent identification of progeroid clinical phenotypes not related to the accumulation of farnesylated lamin A have highlighted that this is not the full explanation for the cellular defects observed in these diseases. Indeed, for example, the majority of APS and AWS cases are caused by different mutations in exons of *LMNA* gene, that do not give rise to splice variants of the protein generating the accumulation of farnesylated products, but are nonetheless characterized by a progeroid phenotype. There are exceptions in which APS and AWS are linked to mutations causing both amino acid changes and the formation of truncated versions of lamin A, that theoretically could remain farnesylated [34,35]. However, its not clear if they contribute to phenotype and amino acid changes seem more important, at least in one case [36], for the pathological phenotype. Along with this, the identification of the causative mutation of NGPS in *BANF1* has further underlined that progeroid laminopathies do not necessarily arise from the accumulation of toxic farnesylated mutant lamin A [18]. Accordingly, cell treatments with farnesyltransferase inhibitors, used to treat HGPS patients, revert only some phenotypic aberrant cellular traits, such as nuclear misshapen, but not all of them. For example, they do not avoid senescence or DNA damage activation in HGPS cells [28]. The same result was obtained by in vivo studies. The treatment with farnesyltransferase inhibitors of two progeria mouse models, ZMPSTE24-deficient mice and LMNA^HG/+^ knock in mice, ameliorates the disease phenotype but not completely, and all treated mice eventually develop severe phenotypes and prematurely die [37–39].

Further evidence supporting the idea that permanent farnesylation is not the only driver in progeria, is the recent finding that a mutant progerin, which is not farnesylated as in HGPS, when expressed in normal cells fully mimics HGPS progerin in its ability to promote senescence [40]. Similar results were obtained also in vivo, in a mouse model expressing a non-farnesylated version of progerin, LMNA^nHG/+^, in which the cysteine of the CaaX motif was replaced with a serine. These animals develop the disease phenotype, although in a milder form if compared to mice expressing farnesylated progerin [38]. The same group reported a second mouse model expressing a different progerin, LMNA^csmHG/+^, that cannot be farnesylated as well, in which a total disease recovery was observed [41], indicating that, actually, which is the importance of permanent farnesylated products in generating pathology is not yet fully clarified.

Altered protein-protein interaction and protein mislocalization or sequestration are now proposed as the origin for the multiplicity of phenotypic traits of progeroid laminopathies. The hypothesis is that an organized lamin A network is necessary for coordinating cellular processes through the formation of macrocomplexes including specific partners. Mutant lamin A networks create abnormal interactions of proteins that act in DNA function, which, in turn, impinges on their correct activity [12,40,42,43]. This hypothesis rests on the discovery that lamin A mutants and, in particular, progerin, differentially interact with a subset of lamin A interacting proteins, and this could account for the deregulation of important cellular processes coordinated by lamins [43–46]. For example, progerin shows an altered interaction with transcription factors including Prx1 and Ing1. It induces their mislocalization and this contributes to the HGPS phenotype [15,46,47]. Progerin was also proven to be unable to interact with members of NURD chromatin remodeling complex, which was shown to contribute to chromatin defects observed in HGPS [21]. Differential interactions of progeroid cells were also observed for DNA repair proteins. Among these, DNA-PKcs, Ku80 and Ku70[48], which interact with lamin A, lose their interaction with progerin. This result indicates that lamin A is functional to the maintenance of a nucleoplasmic pool of DNA repair proteins, which is expected to facilitate their rapid recruitment to sites of DNA damage. The inability of progerin to bind them causes DNA repair impairment, leading to genomic instability [48]. Recently it was demonstrated that progerin interacts preferentially with a pivotal element of the DNA replication machinery, the proliferating cell nuclear antigen (PCNA), which clamps DNA at the forks and favors the processivity of DNA replication [49]. Differently from wild type lamin A, progerin sequesters PCNA far away from replicative forks, causing fork stalling and consequent DNA damage [40,50]. PCNA mislocalization, altering proper DNA replication, accounts for multiple cellular defects of progeroid cells including persistent DNA repair activation and genomic instability [40,50].

The accumulation of different forms, both non farnesylated and farnesylated, of prelamin A, a part from progerin, to toxic level is associated to MADA, MADB and RD progeroid laminopathies and has been also reported in association with cellular stress and senescence [51]. Recent studies analyzing the molecular basis of prelamin A-related chromatin organization changes observed in patients cells, further support the idea that aberrant lamin A could create abnormal interactions of proteins that could affect genome stability. Indeed it was demonstrated that BAF1 interacts preferentially with prelamin A rather than with mature lamin A and this interaction affects BAF1 cellular localization inducing its nuclear retention when prelamin A is abnormally accumulated. This, in turn, induces mislocalization of other chromatin remodeling factors altering the chromatin status [52]. Moreover prelamin A-BAF1 interaction is compromised by the *BANF1* gene mutation occurring in NGPS and this could account, at least in part, to the pathological cellular phenotype [53]. Prelamin A is also able to interact with PCNA better than mature lamin A. Indeed, it was demonstrated that the presence of prelamin A interferes with DNA replication fork stability by sequestering PCNA away from its canonical interaction with lamin A [54].

These studies taken together indicate that progeroid defects are not only linked to farnesylation, but also to the alteration of macrocomplex formation caused by dysfunctional lamin A.

## DNA replication is altered in progeroid syndromes

Different lines of evidence support the hypothesis that a prominent source of DNA damage in progeroid samples is DNA replication impairment. It has been observed that in patients’ HGPS cells and in ectopically expressing progerin cells the S-phase is prolonged, and DNA damage occurs predominantly during this phase [40]. Moreover, γH2AX foci were identified as co-localizing with phospho-RPA32 (Ser33), which is a marker of DNA replication stalled forks [50]. It was also assessed that DNA damage foci are positive for MCM7, which is a component of the DNA replication complex, remaining on site when forks are stalled or collapsed. Finally, as anticipated above, DNA replication impairment in HGPS has been linked to PCNA activity. More specifically, it has been demonstrated that progerin sequesters PCNA, creating PCNA positive intracellular aggregates which localize away from replicating DNA, this causes processivity defects, indicated by the decreased rate of replication fork progression observed in HGPS cells, along with replication stress and consequent DSBs formation [40,50].

Several groups have focused on the molecular dissection of the link between lamins and DNA replication. A first elementary aspect underlining this connection is that lamins are present at replication sites early in S-phase. Another direct relation is that lamins interact with DNA polδ and ϵ [55]. Moreover, A-type lamins are required for the elongation stage of replication [56,57]. In fact, the disruption of the lamina, obtained by using a dominant negative lamin A mutant, induces not only the mislocalization of PCNA but also that of the Replication Factor C (RFC) complex, which as PCNA, is essential for the elongation phase of DNA replication [56,57]. A-type lamins are also required for the resolution of stalled replication forks [58]. Lamin A/C depleted cells are indeed unable to restart forks after a replication stress resulting in shorter track length and in the formation of chromosomal aberrations [58]. Biochemical evidences further support the link between lamins and the replication machinery. In fact, a direct interaction between lamins and PCNA occurs through their highly conserved Ig-fold domain and this interaction is important for PCNA positioning on chromatin [59].

The relation between DNA replication and progerias is supported also from studies of progeroid syndromes caused by mutations in DNA replication genes. For example, mutations in *SPRTN*, encoding the PCNA interacting protein Spartan, with a role in the error-prone translesional DNA synthesis (TLS) [60], were identified in three AWS patients [61]. Spartan dysfunction leads to sustained DNA replication stress characterized by decreased DNA replication fork progression speed, incomplete DNA replication and impaired lesion bypass. These replication defects cause DNA damage that, in turn, induces aging or cancer, due to a leakage of G2/M checkpoint observed in cells from these patients [61,62]. Mice bearing hypomorphic alleles of *SPRTN* are growth retarded, show lipodistrophy, develop cataracts, lordokyphosis and cachexia at a young age [62]. This phenotype recalls those of other progeria models.

We observed progeroid traits in a mouse model defective in a gene implicated in DNA function named *AKTIP* (in humans and *Ft1* in mouse) [63–65]. We described that AKTIP dysfunction causes DNA replication and telomere defects, which in turn generate DNA damage activation and cell senescence [63]. Consistently with the connection of AKTIP both with lamins and DNA function, hypomorphic mice (*Ft1*^kof/kof^, *Ft1* Knock out first) displayed premature aging defects, including reduced body size, lipodistrophy, altered bone density and kyphosis [64]. We demonstrated that the phenotype is partly rescued by co-depletion of p53, pointing to the interplay between DNA damage and progeria. We also defined that AKTIP binds to the replication factors PCNA and RPA70, altogether falling into the triangular connection described here among lamins, progerias and DNA function [65].

A further link between DNA function and lamins comes from studies on mutation of *POLD1*. Heterozygous mutations in *POLD1* have been found in patients with Mandibular hypoplasia displaying Deafness, Progeroid features and Lipodystrophy (MDPL) [66–69]. *POLD1* encodes for the catalytic subunit, called p125 subunit or A unit, of polδ, the polymerase responsible for DNA lagging synthesis also involved in DNA repair [70]. Polδ has been shown to interact with lamin A/C in early S-phase [55]. Currently it is not known which is the impact of *POLD1* mutations at molecular level, but it was suggested that heterozygous mutant *POLD1* leads to increased numbers of stalled replication forks, which would then trigger cellular senescence [68].

## Concluding remarks

Despite the rarity of progeroid syndromes, researches aimed at the understanding of the molecular pathways involved in these diseases are important, not only to find new therapeutic approaches, but also to understand the mechanisms leading to physiological aging. Among progeroid laminopathies the most widely studied is HGPS, caused by mutations leading to accumulation of aberrant lamin A that induces a wide range of pathological phenotypes, both at organismal and cellular level. In HGPS and other laminopathies the large array of aberrant phenotypes reflects the fact that lamins coordinate a variety of fundamental molecular mechanisms, ranging from chromatin organization and regulation, to nuclear structural organization and functioning. The pathogenic molecular mechanism of HGPS was initially attributed to the presence of the farnesyl group in progerin. For this reason therapeutic approaches have aimed at blocking progerin farnesylation. Recently, however, a further important interpretation has emerged from studies on progerias. The hypothesis is that mutated lamins interact differently with their partners creating an aberrant intranuclear macrocomplex scenario. Central processes are affected by this intranuclear misorganization including, importantly, DNA replication and repair. Many players are involved in this aberrant picture, including the DNA processivity factor PCNA.

The identification of DNA replication and genomic instability as drivers of the premature aging phenotype in HGPS and other progeroid diseases will help identifying new therapeutic approaches for these devastating pathologies. In addition, considering that progeroid syndromes recapitulate aspects of physiological aging, the dissection of the mediators of the disease phenotype will also give hints into the coveted comprehension of the complex, multifaceted process of aging in humans.
